# Population pharmacokinetics model for escitalopram in Chinese psychiatric patients: effect of CYP2C19 and age

**DOI:** 10.3389/fphar.2022.964758

**Published:** 2022-07-18

**Authors:** Shujing Liu, Tao Xiao, Shanqing Huang, Xiaolin Li, Wan Kong, Ye Yang, Zi Zhang, Xiaojia Ni, Haoyang Lu, Ming Zhang, Dewei Shang, Yuguan Wen

**Affiliations:** ^1^ Department of Pharmacy, The Affiliated Brain Hospital of Guangzhou Medical University, Guangzhou, China; ^2^ Guangdong Engineering Technology Research Center for Translational Medicine of Mental Disorders, Guangzhou, China

**Keywords:** CYP2C19 genotype, elderly, escitalopam, adolescent, population pharmacokinetics

## Abstract

**Objective:** To establish a population pharmacokinetic model in Chinese psychiatric patients to characterize escitalopram pharmacokinetic profile to identify factors influencing drug exposure, and through simulation to compare the results with the established therapeutic reference range.

**Methods:** Demographic information, dosing regimen, CYP2C19 genotype, concomitant medications, and liver and kidney function indicators were retrospectively collected for inpatients taking escitalopram with therapeutic drug monitoring from 2018 to 2021. Nonlinear mixed-effects modeling was used to model the pharmacokinetic characteristics of escitalopram. Goodness-of-fit plots, bootstrapping, and normalized prediction distribution errors were used to evaluate the model. Simulation for different dosing regimens was based on the final estimations.

**Results:** The study comprised 106 patients and 337 measurements of serum sample. A structural model with one compartment with first-order absorption and elimination described the data adequately. The population-estimated apparent volume of distribution and apparent clearance were 815 and 16.3 L/h, respectively. Age and CYP2C19 phenotype had a significant effect on the apparent clearance (CL/F). CL/F of escitalopram decreased with increased age, and CL/F of poor metabolizer patients was significantly lower than in extensive and immediate metabolizer patients. The final model-based simulation showed that the daily dose of adolescents with poor metabolizer might be as high as 15 mg or 20 mg and referring to the therapeutic range for adults may result in overdose and a high risk of adverse effects in older patients.

**Conclusion:** A population pharmacokinetics model of escitalopram was successfully created for the Chinese population. Depending on the age of the patients, CYP2C19 genotype and serum drug concentrations throughout treatment are required for adequate individualization of dosing regimens. When developing a regimen for older patients, especially those who are poor metabolizers, vigilance is required.

## Introduction

Depression and anxiety disorders affect a large number of people worldwide, which is placing an increasing burden on health services (Hazell, 2021). Nowadays, approaches to treatment include antidepressant and mood-stabilizing drugs, psychotherapy, and physical activity. Escitalopram is still the antidepressant of choice because of its safety, efficacy and tolerability (Cipriani et al., 2009; Sanchez et al., 2014; Cipriani et al., 2018). Escitalopram is highly selective for serotonin transporters and is active against depression (Burke et al., 2002; Wade et al., 2002; Rapaport et al., 2004) and anxiety disorders (Stahl et al., 2003; Davidson et al., 2004).

Escitalopram is an active S-enantiomer of citalopram and is one of the most commonly prescribed selective serotonin reuptake inhibitors (SSRIs). It was launched in the United States in 2002 and China in 2006. The pharmacokinetic profile of escitalopram has been studied extensively in healthy people. The maximum concentration of escitalopram is reached ∼4 h after oral administration of 10–20 mg/day, with an elimination half-life (t_1/2_) of ∼30 h. This supports the therapeutic plan of a once-daily dose of 10–20 mg, and escitalopram is characterized by oral clearance and volume of distribution of 0.48 L/h/kg and 18.3L/kg, respectively (Søgaard et al., 2005; Rao, 2007). Escitalopram is primarily metabolized in the liver by cytochrome P (CYP)450, particularly CYP2C19, which is a highly polymorphic enzyme that causes interindividual pharmacokinetic differences (Rao, 2007; Pastoor and Gobburu, 2014), and is excreted mainly through the kidneys. The effect of age (Dolder et al., 2010; Yang and Scott, 2010), gender (Montejo et al., 2015), smoking (Oliveira et al., 2017; Scherf-Clavel et al., 2019), CYP2C19 phenotype (Huang et al., 2021), hepatic impairment (Areberg et al., 2006), and renal impairment (Dolder et al., 2010) on the pharmacokinetics of escitalopram have been investigated. The findings of these studies were instrumental in developing specific dosing recommendations for escitalopram for specific populations (Hicks et al., 2015; Brouwer et al., 2021). Escitalopram has been approved for use in China for 16 years, and it is the first-line antidepressant medication in China (Rao, 2007). As a result, it is necessary to investigate the factors that may affect the pharmacokinetics of escitalopram in the Chinese population in order to provide a basis for individualized medication in China.

Population pharmacokinetics (PopPK) modeling is a widely used tool to analyze pharmacokinetic data to individualize dosing regimens. Based on this approach, we can identify potential covariates that influence the pharmacokinetics of escitalopram and establish formulas to describe individual parameters. Compared with traditional pharmacokinetics, the advantage of PopPK is that the sparse blood drug concentrations can be used to quantify the intrinsic and extrinsic factors influencing pharmacokinetics by incorporating different covariates. There have been several studies on the PopPK of escitalopram. The PK parameters have been compared in HIV-infected and uninfected psychiatric patients (Courlet et al., 2019). A PopPK model of escitalopram in patients during the perinatal period has been established (Weisskopf et al., 2020). The effect of age, weight, gender and CYP2C19 genotype on escitalopram exposure has been studied in American and Italian patients. (Jin et al., 2010; Akil et al., 2016; Kim et al., 2021). No systematic PopPK analysis of escitalopram has been established in Chinese psychiatric patients. A PopPK/PD model has been developed in Korean healthy volunteers (Kim et al., 2021). Although the mutation frequency of CYP2C19 genotype in the Chinese population was similar to that in Korean population (Dorji et al., 2019), they did not investigate the effect of CYP2C19 genotype. Additionally, CYP2C19 *2 and *3 have much less mutation frequency in European than in East Asian population, but *17 is higher than in East Asian. Therefore, because of the difference in race and CYP2C19 variant allele frequency, investigation in the Chinese population is curial.

In the present study, we established a PopPK model of escitalopram in Chinese psychiatric patients by retrospectively collecting serum drug concentrations and related information. Compared to previous studies, in addition to the influence of age, sex, weight, height, body mass index (BMI) and CYP2C19 genotype, we included liver and kidney function-related biochemical indicators and combination therapy to complete a comprehensive pharmacokinetic evaluation of escitalopram. Simulations were also conducted to investigate whether patients needed to take different doses of escitalopram under different circumstances. The objective of the current study was to develop a PopPK model for escitalopram in Chinese psychiatric patients to explore the potential factors that contribute to variability in escitalopram pharmacokinetics. Furthermore, the model served to predict average drug exposure under various influencing factors through simulation and compared it with the established therapeutic reference range.

## Methods

### Subjects and data collection

The data were obtained from psychiatric inpatients in the Affiliated Brain Hospital of Guangzhou Medical University from 2018 to 2021 and monitored drug blood concentrations during this period. Patients were excluded if there was only one blood concentration measurement, and if there was no reliable information about administration and blood sampling times. This study provided an opportunity to evaluate whether age, sex, weight, height, BMI, smoking, drinking, CYP2C19 genotype, alanine aminotransferase (ALT), mitochondrial aspartate aminotransferase (m-AST), total bilirubin (TBIL), albumin (ALB), urea, serum creatinine (Scr), and combination therapy (such as omeprazole and valproic acid) affected the pharmacokinetics of escitalopram. This study was approved by the Institutional Review Board (IRB) in the Affiliated Brain Hospital of Guangzhou Medical University (Approval number: 2021027).

### Determination of escitalopram concentrations

Blood samples (three to four ml) were collected into coagulation-promoting tubes and centrifuged at 17,600 *g* for 3 min. Serum samples (100 µL) were transferred into 2-ml Eppendorf tubes and mixed with 20 µL internal standard (citalopram-d6) and 500 µL acetonitrile. After vortex-mixing for 10 s and centrifugation at 21,130 *g* for 5 min, ∼100 µL supernatant was removed and transferred to autosampler vials with lining tubing. Escitalopram was measured by HPLC-tandem mass spectrometry (Shimadzu, Kyoto, Japan). Separation was performed on an Agilent Eclipse XDB-C18 column (4.6 × 50 mm, 1.8 µm) with a flow of 0.6 ml/min, and the mobile phase consisted of (A) 75% methanol with 5 mM ammonium formate and (B) methanol for 1.3 min. The injection volume was 1 µL. The linear range was 3–300 ng/ml. This analytical method has been examined by selectivity, specificity, matrix effect, stability, and intra- and inter-batch precision and accuracy.

### Determination of CYP2C19 genotype

DNA was extracted utilizing DNA extraction and purification kits from Shanghai BaiO Technology Co. Ltd. The genotype of CYP2C19 was determined using a human CYP2C19 gene detection kit provided by Wuhan Youzhiyou Medical Technology Co. Ltd. DNA amplification was accomplished after extracting DNA and adding the DNA reaction solution. Following the reaction, the Ct values of various channels were calculated using the amplification curves, and the results were determined. With regard to CYP2C19 isoenzymes, patients were divided into three groups according to the predicted phenotypes: extensive metabolizer (EM) if they were homozygous for the wild-type allele *1/*1; intermediate metabolizer (IM) if they carried the *1/*2 or *1/*3 allele; and poor metabolizer (PM) if they carried the *2/*2 or *2/*3 allele.

### Modeling strategy and software

The PopPK model of escitalopram was created using the nonlinear mixed-effect modeling program (NONMEM, version 7) with the first-order conditional estimation with inter- and intraindividual variability interaction (FOCE-I) method to estimate population parameters and identify candidate covariates. Pirana (version 2.9.0) was used to document and structure model development. Normalized prediction distribution errors (NPDE) test was performed using the NPDE-add on package in R (version 4.1.1). Perl-speaks-NONMEM (version 3.4.2) was used to conduct bootstrap analysis (*n* = 1,000). Goodness-of-fit plots were performed using GraphPad Prism (version 9.1.1). Statistical analysis was performed using SPSS (version 25.0).

### PopPK model development

A basic model without any covariates was developed initially. The pharmacokinetics were described using a first-compartment model with first-order absorption and first-order elimination in terms of apparent oral clearance (CL/F), apparent volume of distribution (V_d_/F), and absorption rate constant (K_a_). Due to the paucity of concentration data within a few hours after oral administration, the absorption phase could not be described. We fixed K_a_ to 0.6 according to an established model in Chinese subjects (Chen et al., 2013). A statistical model was included to describe between-subject and residual variability. The interindividual variabilities of CL/F and V_d_/F were evaluated through an exponential error model (Eq 1), and the intraindividual unexplained variability was through a mixed residual error model (Eq 2).

$$P_{i}=\hat{P}\times e^{\eta_{i}}$$ (1)

Where Pi represents the estimate of *i*th individual parameters (V_d_/F or CL/F), $\hat{\text{P}}$ is the population value of the parameters, and ${\text{η}}_{\text{i}}$ is a random-effects with a mean of zero and variance of ω ^2^ conform to normal distribution.

$$Y=F\times \left(1+\varepsilon_{1}\right)+\varepsilon_{2}$$ (2)

Where Y and F denote the model-observed and -predicted escitalopram concentrations, respectively. $\varepsilon_{1}\;$ and $\varepsilon_{2}$ represents proportional error and additive error, respectively, which follow a normal distribution with a mean of zero and variance of σ ^2^.

The selection of candidate covariates was through the method of stepwise forward selection–backward elimination resulting in the final PopPK model for escitalopram. For concomitant medication, we evaluated the effect of the CYP2C19 inhibitors taken by each patient for that several studies have demonstrated that the magnitude of drug-drug interactions with escitalopram was weak and moderate (Gutierrez et al., 2003; Siccardi et al., 2013) with proton-pump inhibitors having a moderate effect on escitalopram pharmacokinetics (Malling et al., 2005; Gjestad et al., 2015), and we also explored the effect of CYP2C19 inducers. Missing values of weight and height were imputed to the population median value. Covariates would be incorporated into the basic model when their addition reduced the objective function value (OFV) to >6.63 (*p* < 0.01) and removed from the full model when exclusion of the covariates resulted in an increase <10.83 (*p* < 0.001). Eqs 3, 4 were applied for continuous (age, height, weight, etc.) and noncontinuous (sex, CYP2C19 genotype, and combination therapy) covariates, and Eqs 5–7 were used to investigate the influence of CYP2C19 genotypes.

The following were continuous covariates:

$$P_{i}=\hat{P}\times e^{\eta_{i}}\times \left[1+\theta_{\mathrm{COV}}\times \left(\mathrm{Co}v_{i}-\bar{\mathrm{Co}v_{i}}\right)\right]$$ (3)

The following were non-continuous covariates:

$$P_{i}=\hat{P}\times e^{\eta_{i}}\times \left[1+\theta_{\mathrm{COV}}\times \mathrm{CO}V_{i}\right]$$ (4)

Where $\theta_{COV}$ represents the calibrator of parameters, $COV_{i}$ and $\bar{Cov_{i}}$ are the *i*th individual value and population median value of covariates, respectively. For gender covariate, COV = 0 represents male and COV = 1 represents female. The concomitant medication covariate was 0 for patients who did not receive concomitant drugs during escitalopram sampling time, and 1 for patients who received concomitant drugs. CYP2C19*1 encodes the normal function enzyme, and *2 and *3 encode no function. Consequently, homozygous wild-type CYP2C19 *1 had the full drug-metabolizing capacity, and *1/*2 and *1/*3 had reduced metabolism compared to *1/*1. PMs possessed two null alleles, such as *2/*2 and *2/*3, in our analysis. Depending on the phenotype, the CYP2C19 genotype was grouped into three: one for *1/*1 subjects, two for *1/*2 or *1/*3 subjects, and three for *2/*2 or *2/*3 subjects.

$$\text{IF}\;\text{GENE}=1\;\mathrm{CL}=\mathrm{TVCL}\times e^{\eta_{i}}\times \theta_{\mathrm{EM}}$$ (5)

$$\text{IF}\;\text{GENE}=2\;\mathrm{CL}=\mathrm{TVCL}\times e^{\eta_{i}}\times \theta_{\mathrm{IM}}$$ (6)

$$\text{IF}\;\text{GENE}=3\;\mathrm{CL}=\mathrm{TVCL}\times e^{\eta_{i}}\times \theta_{\mathrm{PM}}$$ (7)

### Model evaluation

The precision of parameters and the ability of the final covariate model were assessed by goodness-of-fit plots, bootstrapping, and NPDE. At the same time, the plausibility of estimated parameters and relative standard errors, and changes in both inter- and intraindividual variability were also considered. Goodness-of-fit plots were used for the final model quality evaluation, which included: population predicted concentration versus observed concentrations (as known as dependence variables (DV)); individual predicted concentration versus observed concentrations; population predicted concentrations versus conditional weighted residuals (CWRES); and time after last dose versus CWRES. A bootstrap analysis was performed with resampling 1,000 times. The results of bootstrapping were summarized as median, and 95% confidence intervals of each parameter compared with the corresponding parameters obtained with the origin dataset. NPDE is a model evaluation approach based on the fit of each observation and is not easily influenced by experimental design.

### Simulation

Simulation can provide escitalopram dosing guidance in Chinese psychiatric patients, and it was conducted under several regimens based on the final estimations to find optimal individualized dosing regimens. We predicted steady-state concentration profiles for the therapeutic doses of 5, 10, 15, and 20 mg qd for adolescents ≥12 and <18 years, adults ≥18 and <65 years, and elderly ≥65 years with different CYP2C19 phenotypes (EM, IM and PM). We performed the simulation to establish: 1) whether the steady-state serum levels in adult patients were in the therapeutic range after administration according to the instructions; 2) whether it was necessary to give older and PM patients half the dose of escitalopram; and 3) whether adolescent patients could be administrated the same dosing regimen as adults. Simulation was performed to ensure that >95% of the trough concentrations were within the therapeutic window during therapy.

## Results

### Demographic information

The final dataset for the PopPK model included 106 psychiatric patients and 337 escitalopram measurements in both steady-state and non-steady-state. And the approximate sampling times were most of around trough. All patients were given conventional tablets with 5 mg qd, 10 mg qd, 15 mg qd, 20 mg qd, 5 mg bid, or 10 mg bid. The median dose of escitalopram was 10 mg/day (range 5–30 mg/day). Details on the demographics are summarized in Table 1, and the frequency of CYP2C19 is listed in Table 2. CYP2C19*2, the main mutant and causative allele, was the most common genotype, followed by CYP2C19*3, thus making higher frequencies of *1/*2 and *2/*2 among all test samples. In accordance with the therapeutic drug monitoring guidelines in psychiatry by Arbeitsgemeinschaft für Neuropsychopharmakologie und Pharmakopsychiatrie (AGNP) in 2017 (Hiemke et al., 2018), we collected information on enrolled patients receiving CYP2C19 inhibitors and inducers during the sampling time, as well as drugs with potential effect. Concomitant medications are shown in Table 1. The therapeutic window and laboratory alert level of escitalopram in AGNP guideline were 15–80 ng/ml and 160 ng/ml, respectively (Hiemke et al., 2018). We summarized the blood drug concentration information in Table 3.

**TABLE 1:** Demographic data and patients characteristics.

Characteristics	Median/Number	Range/Ratio
Age (year)	45	12–83
Gender		
Male	59	55.66%
Female	47	44.34%
Weight (kg)	61	37–97
Height (cm)	165	150–180
BMI (kg/m^2^)	22.4	14.87–33.91
Smoking habit		
Yes	3	2.83%
No	103	97.17%
Drinking habit		
Yes	0	0%
No	106	100%
Liver function index		
ALT (U/L)	17	5–162
m-AST (U/L)	4.31	1.51–14.71
TBIL (mg/dl)	9.4	2.6–30.1
Renal function index		
ALB	40.4	30.2–68.2
Urea	3.99	1.69–31.54
Scr	67	30–152
CYP2C19 phenotype		
EM	47	44.34%
IM	49	46.23%
PM	10	9.43%
Concomitant medication		
Omeprazole	6	5.7%
Rifampicin	2	1.9%
Buspirone	11	10.4%
Venlafaxine	1	0.9%
aripiprazole	7	6.6%
Clozapine	13	12.3%
Valproic acid	36	34.0%
Lithium Carbonate	13	12.3%
Diazepam	24	22.6%
Clonazepam	7	6.6%
Olanzapine	39	36.8%
Mirtazapine	11	10.4%
Risperidone	26	24.5%

**TABLE 2:** Allele and Genotype frequencies of CYP2C19.

		Total (N = 106)	Frequency (%)	Phenotype
Allele	*1	143	67.5	Normal
	*2	65	30.6	None
	*3	4	1.9	None
Genotype	*1/*1	47	44.34	Extensive
	*1/*2	48	45.28	Immediate
	*1/*3	1	0.94	Immediate
	*2/*2	7	6.60	Poor
	*2/*3	3	2.84	Poor

**TABLE 3:** Distribution of blood drug concentration in all patients.

Concentration	Number	Ratio (%)
<15 ng/ml	24	7.12
≥15 ng/ml, ≤80 ng/ml	274	81.31
>80 ng/ml, <160 ng/ml	37	10.98
≥160 ng/ml	2	0.59

### PopPK model for escitalopram

The pharmacokinetics of escitalopram were best described by a one-compartment model with first-order absorption and elimination (Courlet et al., 2019; Weisskopf et al., 2020). Owing to the proportional error model that could better describe the present model, we fixed the additive error to 0. The OFV of the basic model was 1923.221. The mean (relative standard error) basic model estimate parameters were 14 L/h (4%) for CL/F and 815 L (16%) for V/F. The between-subject variability was estimated to be 0.146 and 0.216 for CL/F and V/F respectively, and the intraindividual variability was 0.0289 in the proportional error model

A detailed description of the principal results of covariate analyses is presented. During the forward inclusion process, the CYP2C19 phenotype was a significant covariate for CL/F with the model decreased by 25.58 (*p* < 0.001) to a final value of 1897.64. Age also had a significant impact on the CL/F of escitalopram with the value of OFV decreasing by 9.928 (*p* < 0.01) to 1913.293. There were no significant effects of gender, height, weight, BMI, smoking, concomitant medication, and liver or kidney function on CL/F or V/F. When we incorporated age at CL/F forward based on the CYP2C19 phenotype covariate model, the model led to a 21.10 decrease in OFV value to 1876.533, and the full model was developed. The backward elimination step each time removed a covariate from the full model. The values of OFV were increased by 36.76 (*p* < 0.001) and 21.107 (*p* < 0.001) for the CYP2C19 phenotype and age to 1913.293 and 1897.64, respectively, which meant CYP2C19 phenotype and age had significant effect on the exposure of escitalopram. And we found no correlation between age and CYP2C19 phenotype. Estimates for PK parameters of the final model are listed in Table 4.

**TABLE 4:** Final parameter estimates of escitalopram PopPK model.

PK parameters	Final model		Bootstrap	
	Estimate	Rse%	Median	95%CI
Fixed effect				
CL/F (L/h)	16.3	6%	16.4	14.7–18.2
V/F (L)	815	14%	803.9	581.9–1,070.8
K_a_ (h^−1^)	0.6, FIX	—	0.6, FIX	—
θ _Age_	0.0077	20%	0.0077	0.0043–0.0108
θ _IM_	0.847	7%	0.848	0.74–0.97
θ _PM_	0.479	11%	0.478	0.38–0.59
Random effect				
CL/F	0.0877	21%	0.0809	0.0520–0.1254
V/F	0.235	29%	0.215	0.090–0.388
Residual error				
Additive error	0, FIX	—	0, FIX	—
Proportional error	0.0287	12%	0.0288	0.0226–0.0359

**FIGURE 1:**
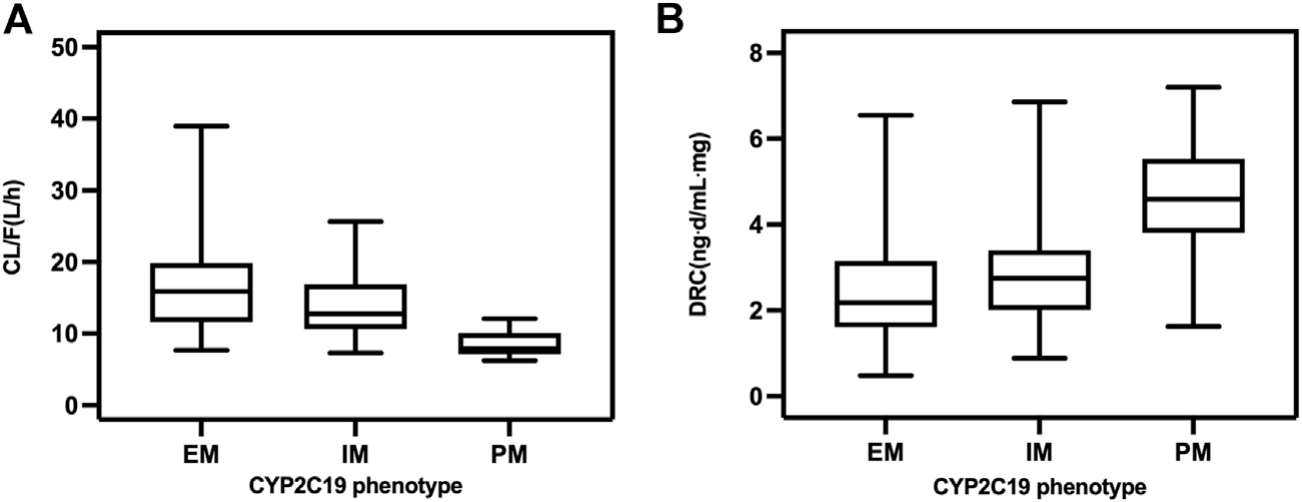
**(A)** CL/F and **(B)** DRC of escitalopram with different CYP2C19 phenotype.

In the final model, there was a decrease in CL/F of escitalopram with increased patient age, and it was also influenced by different CYP2C19 phenotypes. The CL/F was 20.83 L/h in adolescents aged 15 years and 15.84 L/h in adults aged 45 years. In older patients aged 75 years, CL/F decreased to 11.89 L/h. The higher CL/F in EM than in IM and PM patients resulted in the dose-related concentration of IM patients being higher than that in EM patients, while concentration in PM patients was much higher than both IM and EM patients (Figure 1). The estimated population CL/F of escitalopram was 16.73 L/h for EM, 13.96 L/h for IM, and 8.56 L/h for PM patients. CL/F in EM patients was 1.2-fold higher than in IM patients and 1.9-fold higher than in PM patients.

### Model validation

**FIGURE 2:**
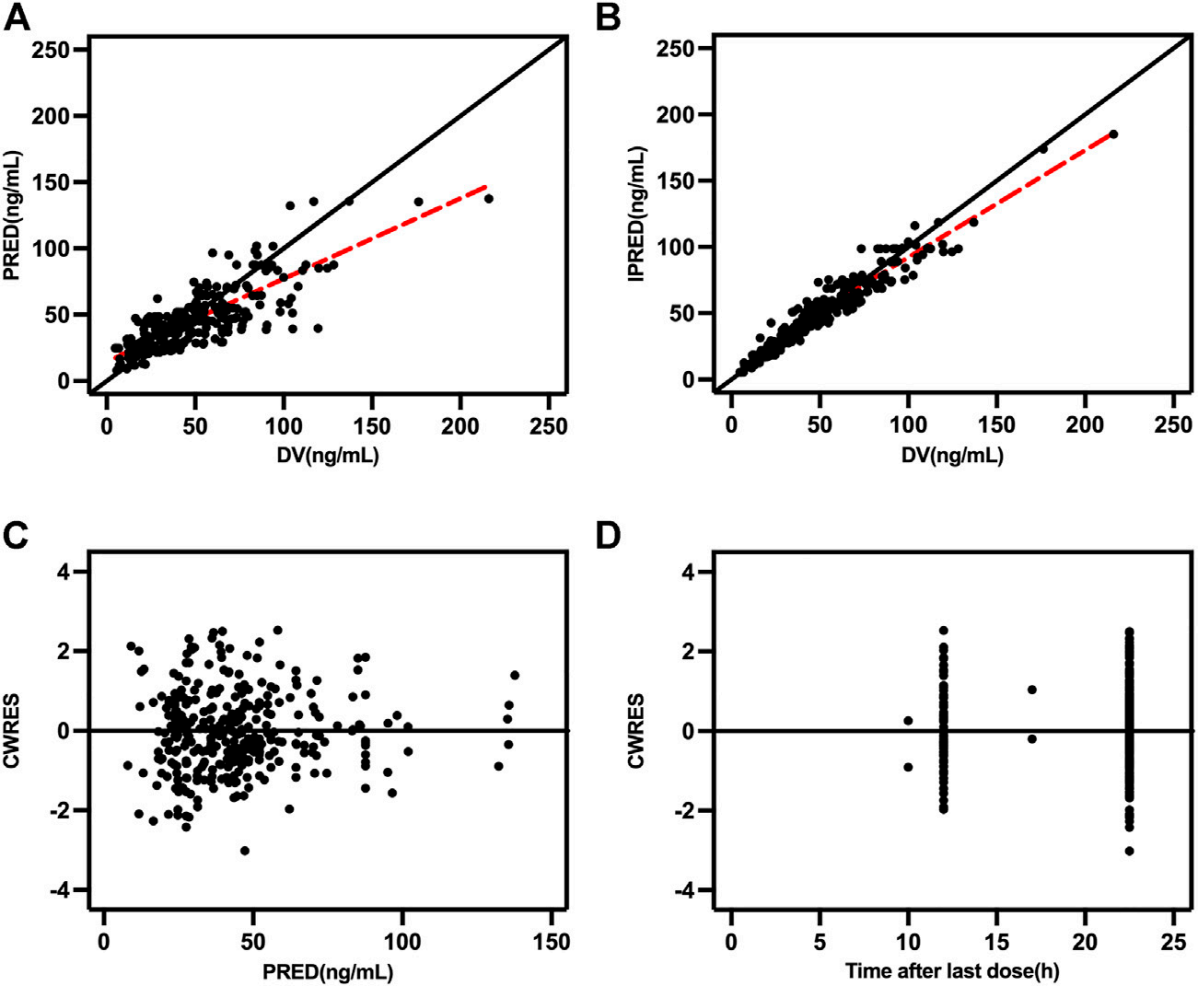
Goodness-of-fit plots **(A)** Population predicted concentration (PRED) versus observed concentrations; **(B)** individual predicted concentration (IPRED) versus observed concentrations; **(C)** population predicted concentrations versus conditional weighted residuals (CWRES); and **(D)** time-after last dose versus CWRES.

**FIGURE 3:**
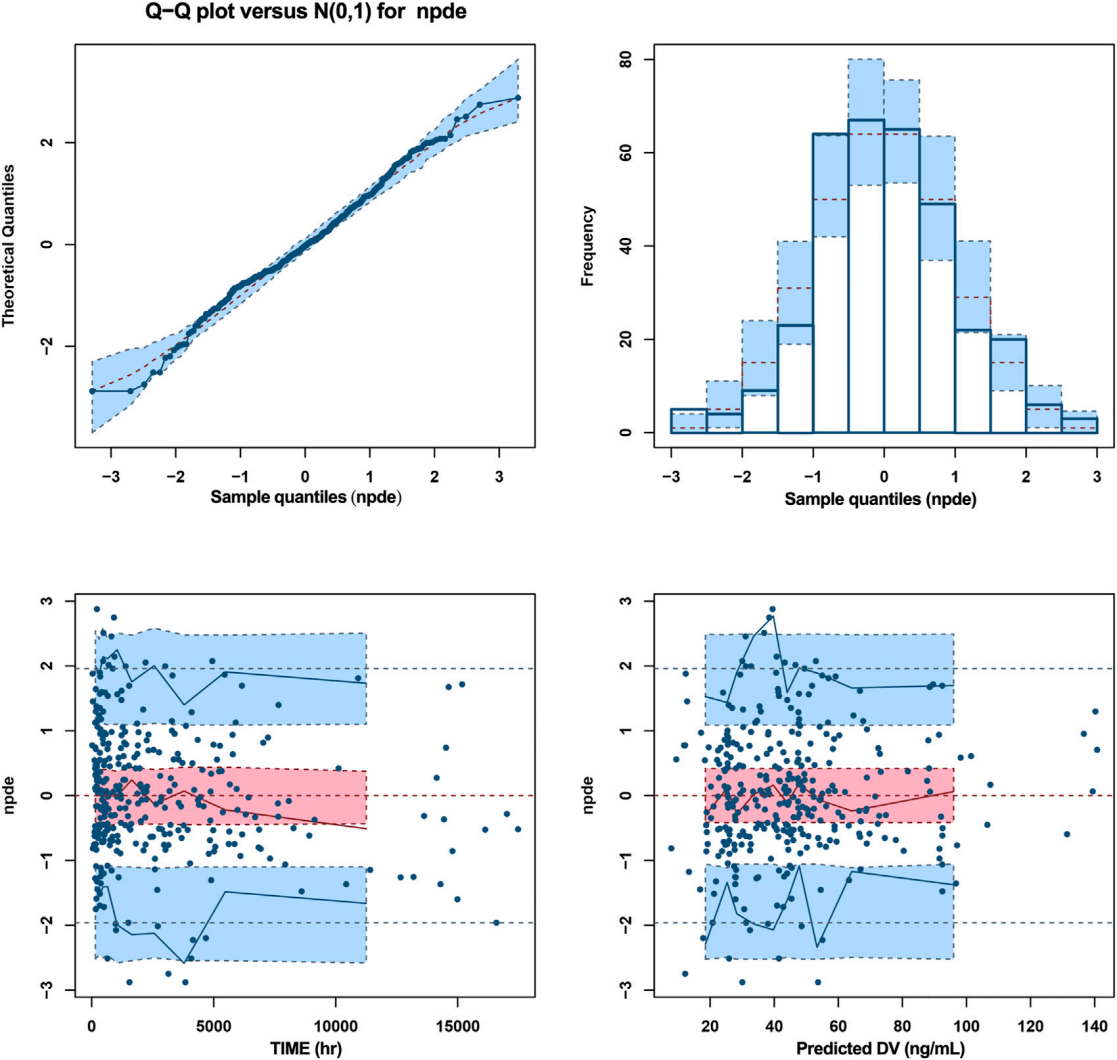
NPDE metrics for the PopPK model of escitalopram. The mean of normalized prediction distribution errors (NPDE) was 0.02359, variance was 0.9894, skewness was 0.04414, and kurtosis was 0.3027. The results of *t*-test and Fisher variance test were 0.664 and 0.911, respectively. The statistical values Shapiro-Wilk (SW) test for normality was 0.0633, and the global adjusted *p*-value was 0.19.

Goodness-of-fit plots, NPDE, and bootstrapping illustrated the appropriateness of the covariate model. Figure 2 showed the scatter plots of the observation values versus population (Figure 2A) and individual (Figure 2B) predicted concentrations, which observed a good correlation and were distributed symmetrically around the trend line. This suggested that the final model was a good fit for the observed data. Figure 2C shows the scatter plots of the CWRES from the final PopPK model, with a range between -3.02 and 2.53, which was distributed symmetrically around 0. The plot of time after dose versus CWRES is shown in Figure 2D. The results of bootstrapping are listed in Table 3. All estimated parameters from the final model were within the 95% confidence interval calculated from the bootstrap method, indicating that the model was constructed with good robustness. The results of NPDE are shown in Figure 3.

### Dosing simulation for escitalopram dose

**FIGURE 4:**
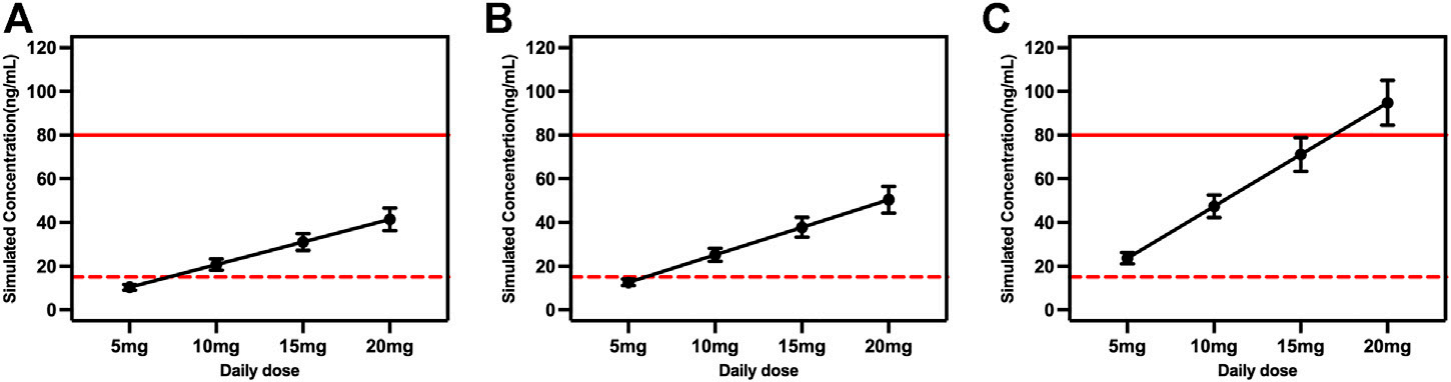
Simulated concentrations for ages ≥18 and <65 years in **(A)** extensive metabolizers; **(B)** immediate metabolizers, and **(C)** poor metabolizers at different daily doses. The red dash lines represented 15 ng/ml, and the red solid lines represented 80 ng/ml.

Considering the covariates that we selected and common situations in clinical practice, the time courses of escitalopram concentrations in steady state were simulated for different ages and CYP2C19 phenotypes. The therapeutic window of escitalopram is 15–80 ng/ml and the laboratory alert level is 160 ng/ml (Hiemke et al., 2018), and applies to patients aged ≥18 and <65 years. Doses of 10, 15 and 20 mg/day were all within the range of 15–80 ng/ml for EM and IM patients (Figures 4A,B). However, the serum drug concentrations were >80 ng/ml at a daily dose of 20 mg for PM patients (Figure 4C).

**FIGURE 5:**
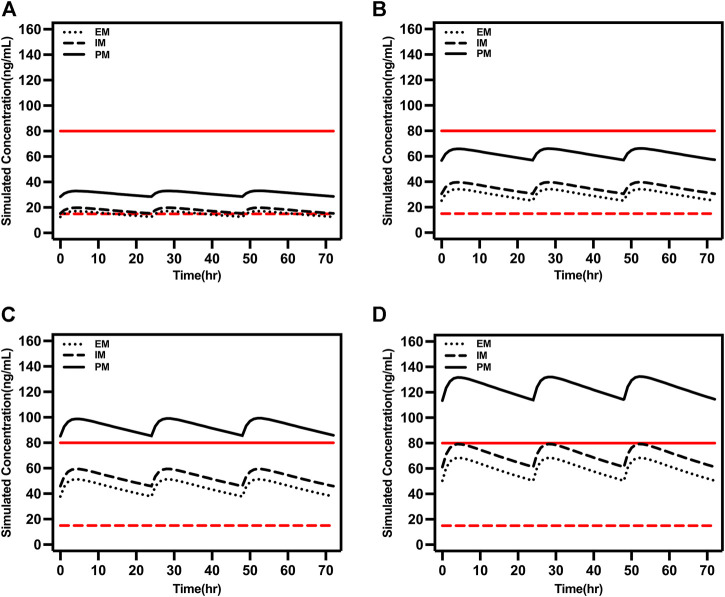
Simulated concentrations in EM, IM, and PM older patients (65 years old) at **(A)** 5 mg/day, **(B)** 10 mg/day, **(C)** 15 mg/day, and **(D)** 20 mg/day. The red dash lines represented 15 ng/ml and the red solid lines represented 80 ng/ml.

The model-based simulation results in older patients showed that the drug concentration in PM patients was twice as high as that in EM patients under the same dosing regimen (Figure 5). Consistent with the above results, oral administration of 15 or 20 mg/day exceeded 80 ng/ml in PM patients. Accordingly, the recommended dose of escitalopram is no more than 10 mg/day for PM patients.

**FIGURE 6:**
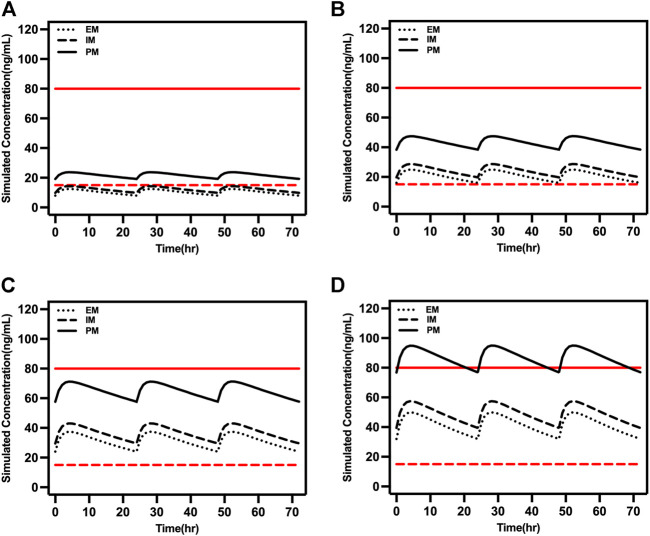
Simulated concentrations in EM, IM, and PM adolescents (16 years old) at **(A)** 5 mg/day, **(B)** 10 mg/day, **(C)** 15 mg/day, and **(D)** 20 mg/day. The red dash lines represented 15 ng/ml and the red solid lines represented 80 ng/ml.

Adolescents typically have higher clearance compared to older people, which was reflected in the steady-state trough concentration being within 15–80 ng/ml when the daily dose was 15 or 20 mg in PM adolescents (Figure 6). However, caution is required for PM patients taking daily doses >10 mg.

## Discussion

PopPK has been utilized extensively in clinical treatment and has become a very useful approach in optimizing individualized dosing regimens, therapeutic drug monitoring, and clinical evaluation of novel drugs. A PopPK model has been created to increase the possibility of meeting suitable pharmacokinetic/pharmacodynamic targets due to the limited therapeutic index of voriconazole and the relatively large systematic interindividual variability (Chen et al., 2019). On the other hand, due to the tendency of order patients to miss doses of medication, they developed a strategy to correct for missed doses through establishing a PopPK model and simulating (Xiao et al., 2021).

In this study, we created a PopPK model for oral administration of escitalopram in Chinese psychiatric patients, while considering demographic, genetic and physiological indicators. The model-predicted covariates of this analysis were in line with several published studies that describe the population pharmacokinetics of escitalopram (Jin et al., 2010; Akil et al., 2016; Courlet et al., 2019). We showed that CL/F of escitalopram varied nearly sevenfold, ranging from 6.26 to 38.93 L/h, which means that the pharmacokinetics of escitalopram in different populations show large interindividual variations. Some intensive sampling designs with escitalopram CL/F of 20–40 L/h, mostly in healthy individuals (Søgaard et al., 2005; Nilausen et al., 2011; Chung et al., 2017), and published PopPK models have a CL/F > 20 or even 30 L/h (van Gorp et al., 2012; Courlet et al., 2019; Kim et al., 2021), while our study showed <20 L/h, which may have been caused by sparse data sampling.

Similar to the previous PopPK studies, our analysis revealed that age and CYP2C19 phenotype contributed differentially to the variability in the pharmacokinetics of escitalopram. Our model results showed a decrease in the CL/F of escitalopram with increasing age. This is in agreement with previously published PopPK models (Jin et al., 2010; Akil et al., 2016). Older patients ≥65 years had a significantly lower CL/F compared with younger healthy volunteers (Fredericson Overø et al., 1985; Bies et al., 2004). As reported previously, older patients had a significantly lower elimination rate than younger patients had (Dhillon et al., 2006), which was confirmed in our study. Actually, our study suggested a 10% decrease in clearance of escitalopram for every 20 years of age, which was less than the previous estimation of a decrease of 30–42% (Jin et al., 2010; Akil et al., 2016). This was consistent with the previously reported decrease in CYP2C19 activity with increasing age (Pollock et al., 1991), and was specifically quantified in our analysis of escitalopram. This might have arisen from the small number of people in each age bracket, although the age ranged from 12 to 83 years. Hence, the dose of escitalopram might need to be adjusted based on age.

In addition, the genetic polymorphism of CYP2C19 had a significant effect on the apparent clearance of escitalopram in previous studies (Areberg et al., 2006; Jin et al., 2010; Chang et al., 2014). Two single-center, randomized, open-label, two-period, two-treatment crossover bioavailability studies with 96 healthy Chinese individuals showed that the exposure of escitalopram in PM subjects and IM subjects increased by 102 and 38% respectively compared with EM, and the efficacy and toxicity of escitalopram varied among individuals with different genotypes (Chang et al., 2014; Jukić et al., 2018). In our study, EM and IM patients with CYP2C19 cleared escitalopram 48.8 and 38.7% faster than PM patients did. This means that metabolism in PMs is greatly reduced, and they experience higher systemic exposure compared with EMs and IMs that have similar clearance. Hence, genetic testing before medication and adjustment of escitalopram dose in PMs should be considered in the clinical treatment of Chinese patients. Moreover, the present findings of the CYP2C19 genotype–phenotype relationship are consistent with the previous study. When breaking the genotype into five categories (*1/*1, *1/*2, *1/*3, *2/*2, and *2/*3), we found that estimations of CL/F for *1/*2 and *1/*3 were similar, as were those for *2/*2 and *2/*3. (Rudberg et al., 2008) showed the effect of CYP2C19*17 on the concentration of escitalopram, and patients with CYP2C19 *17/*17 alleles showed a 42% reduction in concentration. In our study, data for patients with CYP2C19*17 could not be collected because *17 was detected at a low frequency in the Chinese population, and CYP2C19 *1 is the most common allele, followed by *2 and *3 (Chen et al., 2008; Zhou et al., 2009; Tang et al., 2013). We did not consider CYP2D6 and CYP3A4 as covariates because their genetic variation has not been shown to significantly affect serum levels of escitalopram.

We found that sex was not an important factor affecting escitalopram CL/F, although a previous study with a small number of subjects suggested that CL/F of citalopram is higher in men than women (Sidhu et al., 1997). However, sex has not been found to exert a clinically significant effect on pharmacokinetics in healthy volunteers (Søgaard et al., 2005; Dhillon et al., 2006), and (Akil et al., 2016) reported that sex had no effect on escitalopram CL/F. We observed no weight-related difference in escitalopram clearance, although the influence of weight and BMI has been reported previously (Jin et al., 2010; Akil et al., 2016). This may have been caused by incomplete demographic information for some patients enrolled in our study.

Liver and kidney functions affect the metabolism and excretion of drugs. For patients receiving escitalopram with hepatic impairment, the estimated mean area under the curve (AUC) values were 51 and 69% higher for patients with mild and moderate hepatic impairment compared with healthy individuals (Areberg et al., 2006). Although all subjects in the study tolerated the treatment well and no serious adverse events were reported, careful monitoring and dose adjustment during long-term therapy are suggested. There is no conclusive evidence for the role of escitalopram in patients with depression and renal failure; however, pharmacokinetic analysis of citalopram in patients with renal insufficiency revealed that t_1/2_ increased by 35% and renal clearance decreased by 40% (Joffe et al., 1998). Therefore, caution is recommended in such patients when using escitalopram. The data included a small number of patients with liver and kidney impairment; thus, evaluation of the effect of liver and kidney function in this PopPK analysis was limited.

None of the co-ingested drugs interacted pharmacokinetically with escitalopram in our study. (Malling et al., 2005) found that co-administration with cimetidine or omeprazole caused a moderate increase in exposure and t_1/2_, and omeprazole and esomeprazole had a wider effect on escitalopram than sertraline and citalopram had (Gjestad et al., 2015). Proton pump inhibitors are predominantly cleared by CYP2C19. Combination of escitalopram with drugs that are also metabolized by CYP2C19 may produce competitive inhibition between two CYP2C19 substrates. However, there were perhaps only seven of our patients treated with combined escitalopram and omeprazole, and no effect of omeprazole on escitalopram was found. Adjunctive treatment with fluvoxamine significantly increases escitalopram concentration (Yasui-Furukori et al., 2016), but there was no co-administration of fluvoxamine in our patients.

The FDA-recommended initial dose of escitalopram is 10 mg qd in adult patients, and 20 mg qd is the maximum dose. Simulation in our study reveals that the standard 5 mg/day regimen in EM and IM patients may lead to trough concentrations below the therapeutic target of 15 ng/ml, with a risk of suboptimal antidepressant efficacy. We also need to consider the effect of different CYP2C19 phenotypes. The Clinical Pharmacogenetics Implementation Consortium guidelines provide escitalopram dosing recommendations for different CYP2C19 genotypes (Hicks et al., 2015). Despite these guidelines being based on studies on the Caucasian population, they may also be suitable for the Chinese adult population. Consistent with our simulation results, EM or IM patients should initiate therapy with the recommended starting dose and maintenance dose up to 20 mg/day. Although IM patients may have elevated serum concentrations of escitalopram, there is little difference compared to EM patients. For PM patients with lower clearance and higher drug serum levels, the starting dose should be reduced by 50% (5 mg/day) and the maximum maintenance dose is 10 mg/day, or selecting drugs not predominantly metabolized by CYP2C19. Simulation results showed that the steady-state trough concentration was within the therapeutic window at a daily dose of 15 mg, but there is a risk of exceeding 80 ng/ml; thus, the maximum dose of 10 mg is recommended for PM patients, which is consistent with the guidelines. When escitalopram does not reach the target clinical efficacy, an increase in dose to 15 mg can be considered, but blood concentrations and adverse effects should be closely monitored.

Older patients are a special population. Although a single-dose clinical study confirmed that the pharmacokinetics of escitalopram were similar between young and older patients, t_1/2_ and AUC were ∼50% higher than in patients aged 18–35 years. Our study suggests that a daily dose of 10 mg escitalopram gives approximately the same steady-state serum levels in older individuals as a dose of 15 mg in adolescents, and that this is due to the reduced rates of metabolism in the former. Long-term excessive exposure in older people can lead to an increased rate of bradycardia (Barak et al., 2003), falls and fragility fractures (Gorgas et al., 2021); therefore, the starting and maintenance doses need to be fully considered and adjusted by genetic testing and therapeutic drug monitoring. Especially for older PM individuals, trough concentrations are higher than the minimum toxic concentration (80 ng/ml) with 15 or 20 mg/day. The FDA recommends 10 mg as the maximum daily dose for older patients, which implies that referring to the therapeutic range for adults may result in overdose and lead to a high risk of adverse effects. According to the results of the simulation, the therapeutic window on the AGNP guidelines does not extrapolate to people aged ≥65 years and needs to be reformulated. However, it requires to be validated in a large number of clinical trials. The results of the current study can provide a reference for future research.

The FDA approved escitalopram in 2009 for the acute and maintenance treatment of adolescents with major depressive disorder aged 12–17 years. The maximum recommended daily dose for adolescents was 20 mg, which is the same as for adults. Escitalopram was found to be efficacious and well-tolerated in the adolescent population with major depressive disorder when given at a daily dose of 10–20 mg in two clinical trials (Emslie et al., 2009; Findling et al., 2013). The pharmacokinetic differences showed no clinical significance in adolescents compared with adult healthy individuals (Rao, 2007). Furthermore, although the mean t_1/2_ of escitalopram is shorter in adolescents, there are no differences in maximum concentration and AUC (Bareggi et al., 2007), hence the dose regimen was not affected. Our simulation results in adolescents were mostly consistent with those in adults and not significantly influenced by CYP2C19 genotype which was evidenced by serum blood concentrations within the therapeutic window at 15 mg/day and 20 mg/day for PM subjects. However, the efficacy and tolerance needed further investigation. Nevertheless, the risk of manic conversion during antidepressant treatment is highest in patients aged 10–14 years (Martin et al., 2004). Monitoring for suicidality during pharmacotherapy is necessary, and the frequency of monitoring based on each patient’s particular risk.

There were several limitations that need to be considered. First, the sample size was small and most of the samples were at trough concentrations, which did not sufficiently reflect the absorption and distribution characteristics of escitalopram. Second, the small number of PM patients may have been related to the low frequency of mutations, which needs to be confirmed in further studies. Third, there were few cases of combined medication in our analysis, so it will be necessary to explore other drugs that might affect the pharmacokinetics of escitalopram. Notwithstanding, we obtained systematic data to develop a PopPK model in Chinese psychiatric patients for the first time and performed a simulation. These results provide guidance for making a better therapeutic decision on escitalopram dosing regimen to minimize excessively high exposure to this selective serotonin reuptake inhibitor through incorporating age and CYP2C19 genotype into this assessment.

## Conclusion

Our PopPK model demonstrated the influence of age and CYP2C19 phenotype on escitalopram pharmacokinetics in Chinese psychiatric patients. Using a one-compartment model with first-order absorption and elimination achieved good predictive power. According to the simulation results, in contrast to patients ≥18 years, the daily dose for adolescents with PM might be as high as 15 mg or 20 mg and the current therapeutic window of escitalopram might not be suitable for older patients, both of which required further study. Our results emphasized the necessity for genetic testing and therapeutic drug monitoring during treatment for optimal dosage regimen individualization.

## Data Availability

The original contributions presented in the study are included in the article/supplementary material further inquiries can be directed to the corresponding authors.
